# Improved nutritional outcomes with neurally adjusted ventilatory assist (NAVA) in premature infants: a single tertiary neonatal unit’s experience

**DOI:** 10.1007/s00431-022-04411-0

**Published:** 2022-02-22

**Authors:** Kerri Benn, Laura De Rooy, Peter Cornuaud, Anay Kulkarni, Sandeep Shetty

**Affiliations:** 1 Neonatal Unit, St. George’s Hospital NHS Foundation Trust, London, UK; 2 St. George’s University of London, London, UK

**Keywords:** Neurally adjusted ventilatory assist, Prematurity, Neonatal trigger ventilation, Preterm nutritional outcome: Discharge z scores

## Abstract

During neurally adjusted ventilatory assist (NAVA)/non-invasive (NIV) NAVA, a modified nasogastric feeding tube with electrodes, monitors the electrical activity of the diaphragm (Edi). The Edi waveform determines the delivered pressure from the ventilator. Infant breathing is in synchrony with the ventilator and therefore is more comfortable with less work of breathing. Our aim was to determine if infants on NAVA had improved nutritional outcomes compared to infants managed on conventional respiratory support. A retrospective study was undertaken. Infants on NAVA were matched with two conventionally ventilated controls by gestational age, birth weight, sex, antenatal steroid exposure, and whether inborn or transferred ex utero. NAVA/NIV-NAVA was delivered by the SERVO-n® Maquet Getinge group ventilator. Conventional ventilation included pressure and volume control ventilation, and non-invasive ventilation included nasal intermittent positive pressure ventilation, triggered biphasic positive airway pressure, continuous positive airway pressure and heated humidified high flow oxygen. The measured outcome was discharge weight z scores. Eighteen “NAVA” infants with median gestational age (GA) of 25.3 (23.6–27.1) weeks and birth weight (BW) of 765 (580–1060) grams were compared with 36 controls with GA 25.2 (23.4–28) weeks (*p* = 0.727) and BW 743 (560–1050) grams (*p* = 0.727). There was no significant difference in the rates of postnatal steroids (61% versus 36% *p* = 0.093), necrotising enterocolitis (22% versus 11% *p* = 0.279) in the NAVA/NIV NAVA compared to the control group. There were slightly more infants who were breastfed at discharge in the NAVA/NIV NAVA group compared to controls: breast feeds (77.8% versus 58.3%), formula feeds (11.1% versus 30.6%), and mixed feeds (11.1% versus 11.1%), but this difference was not significant (*p* = 0.275). There was no significant difference in the birth z scores 0.235 (−1.56 to 1.71) versus −0.05 (−1.51 to −1.02) (*p* = 0.248) between the groups. However, the discharge z score was significantly in favour of the NAVA/NIV-NAVA group: −1.22 (−2.66 to −0.12) versus −2.17 (−3.79 to −0.24) in the control group (*p* = 0.033).

*Conclusion:* The combination of NAVA/NIV-NAVA compared to conventional invasive and non-invasive modes may contribute to improved nutritional outcomes in premature infants.

**What is known about this topic:**

*•* *Neurally adjusted ventilatory assist (NAVA) ventilation enables synchronisation of both the start and end of an inflation breath and allows the neonate to initiate their own breath and regulate.*

**What this study adds: **

*•* *NAVA when offered to extremely premature infants may have additional benefit of improved nutritional outcomes. *

## Introduction

NAVA is a mode of ventilation (either invasive or non-invasive) that is characterised by a ventilator triggering and assisting in spontaneous breathing based on the patient’s diaphragmatic muscle electrical activity (Edi) [1]. In spontaneous breathing, the respiratory centre in the brainstem generates an electrical signal that is transmitted along the phrenic nerve to stimulate the diaphragm [2, 3]. The diaphragm contracts when stimulated which results in the expansion of the chest wall, leading to negative pressure in the pleural cavity which allows air to be drawn in [1]. In NAVA ventilation, the ventilator delivers a pressure that is synchronised and in proportion to the infant’s diaphragmatic electrical activity (Edi) [4]. The Edi, measured in microvolts (mV), is detected by electrodes embedded into a modified nasogastric tube [2, 3]. The intensity of the Edi signal determines the amount of peak inspiratory pressure (PIP) delivered by the ventilator, which continues until the Edi decreases by 70% [1, 5]. NAVA ventilation therefore enables the synchronisation of both the start and end of an inflation breath [5] and allows the neonate to initiate their own breath as well as regulating their PIP, mean airway pressure, tidal volume and inspiratory times (Ti) [5, 6].

There have been multiple studies demonstrating improved patient-ventilator interactions for NAVA ventilation. In 2018, a prospective observational crossover study done comparing NAVA and conventional ventilation in 23 infants (median GA 27 weeks and median BW of 780 g) showed a significant improvement in patient-ventilator interaction with a lower NeuroSync index for the NAVA cohort (*p* value < 0.05) indicating less asynchrony with NAVA ventilation [7]. Another randomised observational study (2017) illustrated both subjectively and in measuring infants movements that the infants appeared to be comfortable on NIV-NAVA (p = 0.001) [6]. With the infant breathing in synchrony with the ventilator and therefore being more comfortable with less work of breathing, we hypothesised that infants on NAVA would have improved weight gain compared to infants managed on conventional respiratory support.

The aim of our study was to assess whether NAVA followed by NIV-NAVA improved the weight gain in premature infants compared to infants who received conventional ventilation.

## Materials and methods

A retrospective study was undertaken. NAVA mode of ventilation was introduced in the neonatal unit at St Georges from June 2019. Premature infants born less than 32 weeks of gestational age with evolving or established BPD were offered NAVA mode of ventilation at the discretion of the attending neonatal consultant who used local guidance on the use of NAVA ventilation. Eighteen consecutive infants who received NAVA/NIV-NAVA mode of ventilation during the course of invasive and non-invasive ventilation between June 2019 and August 2020 were compared to a historical cohort born between February 2016 and February 2019. The historical cohort was matched by gestational age, birth weight, sex, antenatal steroid exposure and if inborn or transferred ex utero. Infants who had major congenital anomalies were excluded from the study. This project was registered with St. George’s University Hospitals NHS Foundation Trust (SGH) Audit department.

Premature infants born less than 32 weeks of gestational age requiring invasive ventilation support beyond the second week (14 days) of postnatal age were classified as infants with evolving BPD and premature infants born less than 32 weeks of gestational age requiring respiratory support at 36 weeks corrected gestational age were deemed infants who had established BPD. Conventional ventilation included flow-triggered volume targeted ventilation (VTV), assist control ventilation (ACV) and synchronised intermittent mandatory ventilation (SIMV), invasive modes of ventilation and conventional non-invasive modes such as BiPAP tr (triggered bi-level positive airway pressure), BiPAP (bi-level positive airway pressure, CPAP (continuous positive airway pressure) and HHFNC (heated humidified high-flow nasal cannula oxygen). Each infant who received NAVA/NIV-NAVA and other conventional modes of invasive (VTV, ACV, SIMV) and non-invasive (nasal intermittent positive pressure ventilation (NIPPV, BiPAP, CPAP, HHFNC) was matched with two other infants (controls) supported by conventional invasive and non-invasive ventilation.

Infants were identified from a standardised electronic neonatal database (Badgernet). Data were obtained from the electronic documentation recording system and cross checked with the medical notes. NAVA/NIV-NAVA was delivered by the SERVO-n® Maquet Getinge ventilator. Outcomes were birth z scores, discharge weight and discharge z scores.

Infants were historically routinely supported by the Stephanie ventilator (Stephan GMBH, Gackenbach, Germany). Volume targeted ventilation (VTV), assist control ventilation (ACV), synchronised intermittent mandatory ventilation (SIMV) and non-triggered non-invasive modes including BiPAP tr, BiPAP, CPAP (flow driver) and HHFNC were the standard ventilation modes for infants born prematurely. The standard initial mode of invasive ventilation was AC-VTV for most infants and if the clinical decision was made to change over to pressure mode, then ACV or SIMV was considered based on the blood gases and gestational age of the infant. The standard initial mode of non-invasive ventilation was BiPAPtr/BiPAP or CPAP and changed to HHFNC based on circuit change dates and departmental policy [8]. From June 2019, Stephanie (Stephan GMBH, Gackenbach, Germany) ventilators were transitioned to Maquet Servo N ventilators which are currently the only ventilators which have NAVA ventilation modality. When on NAVA, the apnoea time was set to 2 s and the upper pressure limit is at least 5 cm H_2_O higher than the baseline settings. A six French 49 or 50 cm Edi catheter was inserted via the oro-gastric route and correct positioning confirmed as per the instructions of the manufacturer using the Edi catheter positioning guide function on the ventilator (Maquet Servo-n User Manual Version 4.1). The guide function displays the retrocardiac electrocardiogram (ECG), and correct positioning was confirmed when the P waves and QRS complexes were visible in the uppermost leads and then decreased in size until the P waves disappeared in the lowest lead. Coloured highlighting of the central two leads appeared once the catheter was in the correct place. Once correct positioning was confirmed, the catheter was securely attached to the infant’s face using an adhesive dressing. Before the infant was changed to NAVA mode from conventional ventilation (VTV, ACV, SIMV), the NAVA level was adjusted so that the estimated pressure waveform on NAVA closely matched the actual pressure waveform on the baseline settings, aiming for the peak Edi to be between 5 and 15 µV as per the recommendations of the manufacturer. The baseline ventilator settings were used to determine the back-up settings to be used on NAVA in the absence of an Edi signal. Once weaned to NAVA level of 0.8–1.0 cmH_2_O/mV, infants were extubated onto NIV-NAVA mode on SERVO-n® Maquet Getinge ventilator. The apnoea time was set to 2 s, and the upper pressure limit is at least 5 cm H_2_O higher than the baseline settings.

BPD was defined as need of oxygen at corrected 36 weeks of gestation as per NICHD definition [9].

## Statistical analysis

The anthropometric data of birth weight and discharge weight was converted to z-scores using the Fenton growth charts. The calculation of z-scores is based on a growth reference calculating how many standard deviations the weight is from a mean value for the premature infant [10]. Differences between the two groups were assessed for statistical significance using the chi-square test or the Mann Whitney test as appropriate using IBM SPPS statistical software, V.26 (IBM Corporation, USA).

## Results

Eighteen “NAVA” infants with median gestational age (GA) of 25.3 (23.6–27.1) weeks and birth weight (BW) of 765 (580–1060) grams were compared with 36 controls with GA 25.2 (23.4–28) weeks (*p* = 0.727) and BW 743 (560–1050) grams (*p* = 0.727). Duration of NAVA/NIV was for a median (range) of 14 5–7 days. There was no significant difference in the total duration of invasive ventilation for 27 (14–92) days versus 27 (14–99) days (*p* = 0.614) and non-invasive ventilation for 56 (32–140) days versus 52 (9–145) days (*p* = 0.157) in the NAVA group compared to the historical cohort. There was no significant difference in the rates of BPD (77% versus 66% *p* = 0.532), post-natal steroids (61% versus 36% *p* = 0.093) and necrotising enterocolitis (NEC) (22% versus 11% *p* = 0.279) in the NAVA/NIV NAVA compared to the control group. (Table 1). There was no significant difference in the birth z-scores 0.235 (*−*1.56 to 1.71) versus −0.05 (*−*1.51 to −1.02) *p* = 0.248 in between the groups. However, the discharge z-score was significantly in favour of the NAVA-NIV-NAVA group −1.22 (−2.66 to −0.12) versus −2.17 (−3.79 to −0.24) in the control group (*p* = 0.033) (Table 2).

**Table 1:** Demographic data. Data displayed as median (range) or %

	**NAVA/NIV NAVA (*n* = 18)**	**Control (*n* = 36)**	***p* value**
BW (grams)	765 (580–1060)	742.5 (560–1050)	0.727
GA (weeks)	25.29 (23.6–27.7)	25.14 (23.4–28)	0.727
Gender (male/total)	10/18 (56%)	20/36 (56%)	1
Antenatal steroids (yes/total)	17/18 (94%)	34/36 (94%)	1
In utero/ex utero (in utero/total)	13/18 (72%)	28/36 (78%)	0.74
Overall duration of invasive ventilation (days)	27 (14–92)	27 (14–99)	0.614
Overall duration of NIV (days)	56 (32–140)	52 (9–145)	0.157
Postnatal steroids (yes/total)	11/18 (61%)	13/36 (36%)	0.093
NEC (yes/total)	4/18 (22%)	4/36 (11%)	0.279
BPD (yes/total)	14/18 (77%)	24/36 (66%)	0.532
Type of milk at discharge	Breast 14/18 (77.8%)	21/36 (58.3%)	0.275
	Formula 2/18 (11.1%)	11/36 (30.6%)	
	Mixed 2/18 (11.1%)	4/36 (11.1%)

**Table 2:** Results by ventilatory mode. Data displayed as median (range) or %

	**NAVA/NIV NAVA (*n* = 18)** **Median (range)**	**Control (*n* = 36)** **Median (range)**	***p* value**
Birth Z score	0.235 (−1.56 to 1.71)	0.05 (−1.51 to 1.02)	0.248
Discharge weight (grams)	3066 (2348–5785)	2642.5 (1835–6560)	0.088
Z score at discharge	−1.22 (−2.66 to −0.12)	−2.17 (−3.79 to −0.24)	0.033

## Discussion

We have demonstrated that in infants born very prematurely and with evolving or established BPD, a combination of NAVA/NIV-NAVA compared to conventional invasive and non-invasive modes could lead to improved nutritional outcomes in premature infants. The Rong et al. (2019) study showed that NAVA ventilation appeared to be better tolerated than standard conventional ventilation, with a significant decrease in the need for sedation after switching to NAVA (*p* = 0.012) [11]. However, this study showed no difference in weight gain (g/day) as a secondary outcome [11]. In our work, we have examined the infants’ growth trajectories, as illustrated by their z-score at birth and at discharge. We used the Fenton z-score calculator, derived from the revised 2013 Fenton growth charts [12]. The advantage of using the z-score is that this measure takes into account postnatal age and gender [13]. The main difference between the study group and control group was a significant increase in the discharge weight z-score in the infants who received NAVA or NIV-NAVA ventilation compared to the control group (*p* value 0.033). We hypothesise that a potential cause for the improvement in the growth trajectory is the improved synchronisation and patient-interaction/comfort level with NAVA ventilation, resulting in less work of breathing.

While there was nearly double the use of steroids in the NAVA/NIV NAVA group compared to controls, the growth trajectory of the NAVA group is improved, despite the possible negative effects of steroids on postnatal growth. Also, there was no significant difference between the duration of invasive ventilation days between the NAVA and historical cohort, and as unit policies and guidance as to the use of postnatal steroids had not changed over the years in question, this may however indicate that infants in the NAVA group had greater respiratory requirements.

There are strengths and some limitations to our study. To our knowledge, this is the first single centre study which has demonstrated improved nutritional outcomes in premature infants who received a combination of NAVA and NIV-NAVA. While comparison was made to historical controls which has a potential for bias, they were matched for gestational age, birth weight, sex, antenatal corticosteroid exposure, and whether inborn or transferred ex utero. Of further note is that while the conventional ventilation group was identified from patients admitted between 2016 and 2019 and the NAVA group from 2019 onwards, new nutritional policies had been introduced at this neonatal centre in 2015, with no significant changes during the two epochs.

In conclusion, the work suggests that using NAVA ventilation in premature infants leads to an improvement in their growth trajectory, although further large randomised controlled trials are required to confirm this.

## Data Availability

Data given in tables and as text in manuscript.

## Abbreviations

ACV: Assist control ventilation
BiPAP tr: Triggered bi-level positive airway pressure
BiPAP: Bi-level positive airway pressure
BW: Birth weight
CPAP: Continuous positive airway pressure
Edi: Diaphragmatic muscle electrical activity
GA: Gestational age
HHFNC: Heated humidified high-flow nasal cannula oxygen
NAVA: Neurally adjusted ventilatory assist
NIV: Non-invasive ventilation
mV: Microvolts
SIMV: Synchronised intermittent mandatory ventilation
Ti: Inspiratory times
VTV: Volume targeted ventilation
